# Cases of Gunshot Wound

**Published:** 1862-09

**Authors:** O. B. Ormsby

**Affiliations:** Assistant-Surgeon 18th Reg’t. Ill. Vols.


### ARTICLE XXVIII.

### CASES OF GUNSHOT WOUND.

Reported by O. B. ORMSBY, Assistant-Surgeon 18th Reg't. Ill. Vols.

W., of Stewart's Cavalry, aged 21 years, dark hair, black eyes, robust and in good health, was wounded July 28th, by a shot from a carbine or Maynard rifle, which entered his right shoulder, one and a-half inches below and posterior to the acromion process of the scapula, passed directly through the head of the humerus, and made its exit two and a-half inches below the middle of the clavicle. He was transported about 20 miles in an ambulance to the general hospital at Jackson, Tenn. Upon examination, it appeared that the ball had passed through the head of the bone without producing a great amount of comminution, and it could not be ascertained with certainty that the bone was at all injured by cracking or splitting outside the track of the ball. In the absence of positive assurance of greater injury, it was decided to rely upon the recuperative energies of nature to effect a cure. Resection was spoken of, but not being deemed absolutely necessary, was not performed. He was ordered cold dressings, which were continued without other interference, until July 31st., when he had sulph. magn. ℥ji. to relieve constipation. Cold dressing alone was then resumed, and continued until Aug. 8th, when he had Rochelle salts, ℥ss., which failing to produce action of the bowels was repeated upon the following day. Cold dressing was again resumed until the 11th, when he had sulph. magn, ℥i. in two doses, taken six hours apart. Cold dressing again. On the 14th, was ordered stimulants and generous diet, which was continued up to the 20th, when the discharge had nearly ceased. His bowels being somewhat torpid, and his skin showing evidence of biliary derangement, he had on that day sub. mur. hydrarg. grs. v., at once. 22d. Hydrarg. cum creta, grs. x. Two powders given, six hours apart. 24th. Hydrarg. cum creta, pulv. Doveri, aa. grs. v., M., take at once. 25th, Hydrarg. cum creta, grs. v., at once. 26th. Fluid ext. prunus

Virginiana, ℥ss., at intervals of three hours. 30th. Returns home soon; flesh and appetite good; soreness pretty much all gone. No more discharge of pus, and some motion in the joint. Only one or two small pieces of bone were discharged from the wound during the entire time. Had at first a little irritative fever, which immediately yielded when the bowels were freely opened.

In my opinion, this patient made a more speedy recovery, and will have a more useful limb than could have been secured to him by any operation whatever.

S. L., Irish, aged 38 years, wounded July 29th, by musket shot, which entered near the left scapula, and made its exit between the fourth and fifth ribs, of the same side, about two and a-half inches posterior to the left mamma. Was admitted to the general hospital, Aug. 2d, when there was considerable febrile excitement; pain in the left breast; difficulty of breathing; and occasional cough, with expectoration of blood. The wound was carefully examined, and all foreign substances were removed. The wounds were then dressed with compresses dipped in cold water; and as he complained of slight diarrhœa, was directed to take opium, grs. iii., sub. nit. bismuth, grs. xv., M., Divide into three powders, one every two hours. Aug. 3d. Dress with water. Aug. 4th. No movement of the bowels since the day before yesterday. Pulse 100, strong, tongue coated; give Rochelle salt, ℥js. at once. Complains of much pain and restlessness at night, for which he had morphia sulph. grs. iii., in six parts, to be used at discretion of the nurse. 5th. Salts acted on the bowels, and from this day to the 9th, he had cold dressings and an occasional dose of morphia. On the 9th, the fever was abated, and the patient complains but little; but whenever he coughs, which is frequently, air is driven forcibly through the anterior wound, accompained by bloody serum and pus. He was directed to take pulv. Doveri, grs. vi., at every severe paroxysm of cough; also, to have porter or wine. 10th. Bowels torpid; give Rochelle salt, ℥js.; continue cold dressing. 11th. Bowels have not moved; magn. sulph. ℥ss. 12th. Wine. 13th. Sulph. magn. ℥ss. 14th. Wine. 15th. Brandy toddy. 16th.

Continue toddy, and give Rochelle salt, ℥ss. 17th. Toddy. 18th. Very weak; bowels costive; give sulph. magn. ℥i., whiskey, ℥viii., M., and give ℥ss. every two hours, until the bowels move. 19th and 20th. Toddy. 21st. Has had fever two afternoons in succession; give sulph. quinina, grs. x., aromat s. acid, ʒss., ether sulph. ʒi., aqua, ℥iv., M., tablespoonful every two hours. 22d. Wine. 23d. To-day, the air ceased to pass out through the wound. 24th and 25th. Wine. 26th. Has diarrhœa; takes Hope's mixture, ℥iv., every two hours. 27th. Continued. 28th. Wound in the back is closed. 29th. No more wine; rest, and good diet. Sept. 2d. Needs only a little more flesh to make him fit for duty.

In each of these cases, the sulph. magn., even when it failed to act on the bowels, appeared to remove the febrile excitement almost entirely.

